# Plasmacytoid Dendritic cells (pDCs) in HIV-infected and HIV/HCV-co-infected patients receiving successful treatment

**DOI:** 10.7448/IAS.17.4.19642

**Published:** 2014-11-02

**Authors:** Olga Khokhlova, Ara Reizis, Lidya Serebrovskaya, Natalia Gerasimova, Vadim Pokrovskiy

**Affiliations:** 1 The Central Institute for Epidemiology, Russian Federal Center AIDS, Moscow, Russian Federation; 2 Clinical Department of Infectious Pathology, The Central Institute for Epidemiology, Moscow, Russian Federation

## Abstract

**Introduction:**

Depletion of cellular pool and constant activation of plasmacytoid dendritic cells (pDCs) in HIV-infected persons (HIV+) are associated with disease progression and manifestation of opportunistic infections. The influence of highly-active antiretroviral treatment (HAART) and suppressed HCV-co-infection (HIV+/HCV+) on the extent of these changes remains unknown.

**Objective:**

To study parameters of pDCs in peripheral blood of HIV+ and HIV+/HCV+ patients on HAART.

**Methods:**

Twelve uninfected with HIV or HCV volunteers and 37 patients (12 HIV+, 9 HIV+/HCV+ and 16 HCV+) were enrolled. All HIV+ patients received HAART and had undetermined HIV viral load. All HCV+ patients had finished antiviral therapy (Pegasys/ribavirin) with sustained viral response for six months and more. The pDC population was enumerated by flow cytometry. In vitro IFN production in the whole blood in response to pDC-specific stimulus unmethylated CpG oligonucleotides was determined by enzyme-linked immunosorbent assay (ELISA).

**Results:**

The percentage of pDCs of peripheral blood mononuclear cells (PBMC) in HIV+/HCV+ was the lowest (0.08±0.02) but statistically not different from HIV+ (0.15±0.03; p=0.11) and significantly lower than HCV+ (0.17±0.015; p=0.4) and controls (0.27±0.045; p=0.03). Absolute number of pDCs in HIV+ on HAART (6.3±1.3) was not significantly lower than the control (10.25±1.75; p=0.09) but in HIV+/HCV+ (5.1±1.2; p=0.03), the difference was valid. IFN-alpha production by pDCs in patients on HAART in HIV+ (2.4±0.9 pg/µl) and in HIV+/HCV+ (3.7±1 pg/µl) patients were significantly higher than in controls (in controls IFN-alpha production by pDCs in the native plasma was below the level of detection (3 pg/µl), p=0.02 and p=0.0014).

**Conclusions:**

Absolute number of pDC in HIV-positive person on HAART with sustained viral response to treatment of HCV-co-infection was lower than in HIV-mono-infected. IFN-alpha production by pDC in HIV+ and in HIV+/HCV+ patients receiving HAART remains higher than in uninfected persons.

